# Induction of Apoptosis by Gluconasturtiin-Isothiocyanate (GNST-ITC) in Human Hepatocarcinoma HepG2 Cells and Human Breast Adenocarcinoma MCF-7 Cells

**DOI:** 10.3390/molecules25051240

**Published:** 2020-03-09

**Authors:** Asvinidevi Arumugam, Muhammad Din Ibrahim, Saie Brindha Kntayya, Nooraini Mohd Ain, Renato Iori, Stefania Galletti, Costas Ioannides, Ahmad Faizal Abdull Razis

**Affiliations:** 1 UPM-MAKNA Cancer Research Laboratory, Institute of Bioscience, Universiti Putra Malaysia, UPM Serdang, Selangor 43400, Malaysia; ashvini6050@gmail.com (A.A.); marc_dean89@yahoo.com (M.D.I.); saiebrindhak@yahoo.co.uk (S.B.K.); noorainim@upm.edu.my (N.M.A.); 2 CREA Consiglio per la ricerca in agricoltura e l’analisi dell’economia agraria, Centro di Ricerca Agricoltura e Ambiente (CREA-AA), 40128 Bologna, Italy; renato.iori48@gmail.com (R.I.); stefania.galletti@crea.gov.it (S.G.); 3 Faculty of Health and Medical Sciences, University of Surrey, Guildford, Surrey GU2 7XH, UK; c.ioannides@surrey.ac.uk; 4 Laboratory of Molecular Biomedicine, Institute of Bioscience, Universiti Putra Malaysia, UPM Serdang, Selangor 43400, Malaysia; 5 Laboratory of Food Safety and Food Integrity, Institute of Tropical Agriculture and Food Security, Universiti Putra Malaysia, UPM Serdang, Selangor 43400, Malaysia; 6 Department of Food Science, Faculty of Food Science and Technology, Universiti Putra Malaysia, UPM Serdang, Selangor 43400, Malaysia

**Keywords:** glucosinolate, gluconasturtiin-isothiocyanate, apoptosis, cancer chemoprevention

## Abstract

Gluconasturtiin, a glucosinolate present in watercress, is hydrolysed by myrosinase to form gluconasturtiin-isothiocyanate (GNST-ITC), which has potential chemopreventive effects; however, the underlying mechanisms of action have not been explored, mainly in human cell lines. The purpose of the study is to evaluate the cytotoxicity of GNST-ITC and to further assess its potential to induce apoptosis. GNST-ITC inhibited cell proliferation in both human hepatocarcinoma (HepG2) and human breast adenocarcinoma (MCF-7) cells with IC_50_ values of 7.83 µM and 5.02 µM, respectively. Morphological changes as a result of GNST-ITC-induced apoptosis showed chromatin condensation, nuclear fragmentation, and membrane blebbing. Additionally, Annexin V assay showed proportion of cells in early and late apoptosis upon exposure to GNST-ITC in a time-dependent manner. To delineate the mechanism of apoptosis, cell cycle arrest and expression of caspases were studied. GNST-ITC induced a time-dependent G_2_/M phase arrest, with reduction of 82% and 93% in HepG2 and MCF-7 cell lines, respectively. The same treatment also led to the subsequent expression of caspase-3/7 and -9 in both cells demonstrating mitochondrial-associated cell death. Collectively, these results reveal that GNST-ITC can inhibit cell proliferation and can induce cell death in HepG2 and MCF-7 cancer cells via apoptosis, highlighting its potential development as an anticancer agent.

## 1. Introduction

Chemoprevention is a cancer-control strategy to intervene, delay, or halt carcinogenesis by administration of naturally occurring or synthetic chemicals compounds. Chemoprevention is highly regarded as a prospective anticancer approach to counteract cancer incidence and mortality [1]. Epidemiological and numerous clinical studies have provided the basis for cancer therapy via dietary resources [2,3]. Induction of apoptosis is considered one of the primary mechanisms employed by natural chemopreventive agents [4,5,6]. Apoptosis, or programmed cell death, is a tightly regulated cellular suicide program in which individual cells are destroyed while the integrity and architecture of surrounding tissue is preserved [6]. Evasion of apoptosis is a common hallmark of cancer cells, and consequently, most current anticancer therapies are targeted to facilitate the cell death of cancer cells [7].

Indeed, a wide range of natural compounds have been identified in routinely consumed vegetables with potential inhibitory effect on carcinogenesis. These compounds include glucosinolates (GLs), sulfur-containing compounds that act as precursors for the formation of various isothiocyanates (ITCs) [8]. GLs, which are generally not biologically active, are stored in the plant cell vacuole and are released upon disruption of plant tissue and hydrolyzed by the enzyme myrosinase to form a diverse range of potential allelochemicals including ITCs [9]. ITCs are noticeably present as GL precursors in various cruciferous vegetables of nutritional interest including broccoli, cabbage, watercress, cauliflower, mustard, and radish and accounts for the anticarcinogenic activity associated with these vegetables [10]. It has now been established that isothiocyanates such as sulforaphane act as potent cancer chemopreventive agents through a number of mechanisms. For example, isothiocyanates can prevent the formation of the reactive metabolites of chemical carcinogens that interact with DNA by modulating cytochrome P450 and phase II enzymes [10] and can impair cellular proliferation [6]. An additional principal mechanism responsible for their chemopreventive activity is to stimulate apoptosis, resulting in the removal of cancer cells. Indeed, studies with sulforaphane in a number of systems have established that this isothiocyanate elevates apoptotic activity [5].

**Figure 1:**
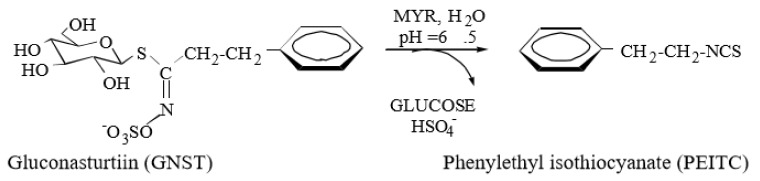
Hydrolysis of gluconasturtiin to phenethyl-isothiocyanate (PEITC) catalyzed by myrosinase at neutral pH. Adapted from Barba et al. [9].

As such, phenethyl-isothiocyanate (PEITC) is one of the most studied bioactive ITCs (Figure 1) that has received increasing attention over the last few decades due to its chemopreventive capacity [10,11]. PEITC is consumed daily in the form of its GL precursor gluconasturtiin (GNST) through dietary intake, with watercress (*Nasturtium officinale*) being one of its principal sources [12]. Myrosinase-catalysed hydrolysis of GNST yields PEITC [13].

Earlier studies have revealed that ITCs manifest their chemopreventive activity, at least partly, via regulating the processes of apoptosis [8] and cell proliferation in human malignant melanoma cells [14]. In the present study, glucosinolate and myrosinase were added together to the incubation system to mimic human exposure since humans largely consume glucosinolates, which are degraded to the isothiocyanate by plant and intestinal myrosinase. The amount of myrosinase added was in excess so that the isothiocyanate is immediately released. Both cells were also chosen due to the risk factors of breast cancer patients predisposed to liver metastases [15], which is known as breast cancer liver metastases (BCLM).

## 2. Results

### 2.1. GNST-ITC-Mediated Cytotoxicity

A decrease in cell viability was observed in both HepG2 and MCF-7 cell lines when treated with GNST (0.1–100 µM) with and without the presence of myrosinase from 24 to 72 h, with the latter cell line being more susceptible (data not shown). In contrast, no decrease in cell population was observed in both HepG2 and MCF-7 cell lines when treated with GNST only. GNST-ITC induced cytotoxicity in both HepG2 and MCF-7 cells with IC_50_ values of 7.83 µM and 5.02 µM, respectively, after 72 h of incubation (Table 1). These concentrations were further used to elucidate induction of apoptosis by GNST-ITC.

### 2.2. GNST-ITC-Mediated Morphological Changes in HepG2 and MCF-7 Cells

**Figure 2:**
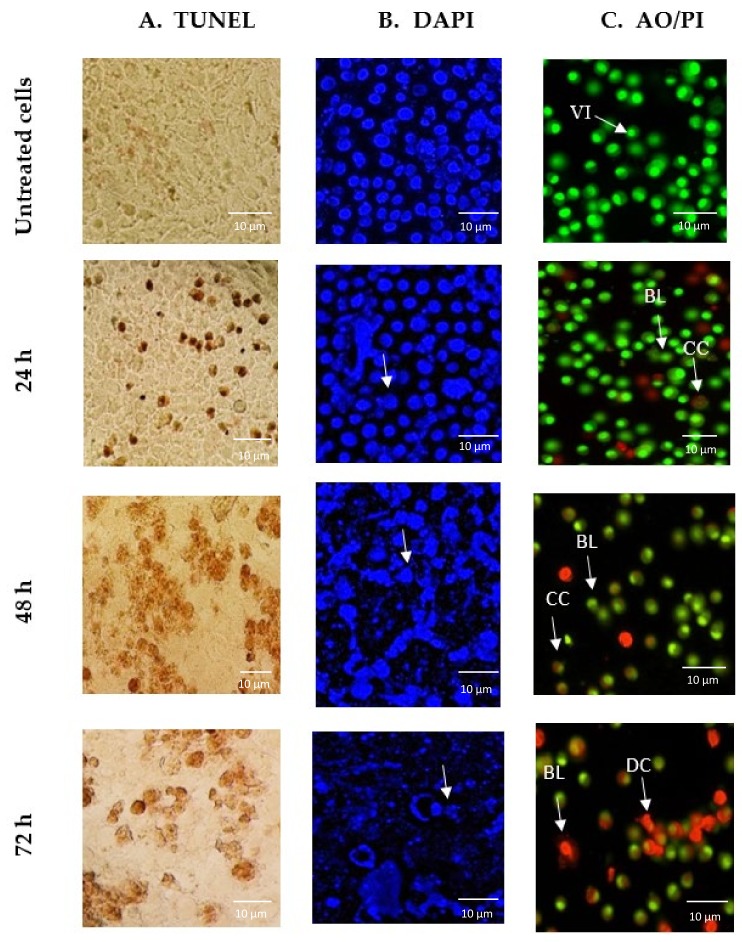
Images of GNST-ITC-induced cell death in HepG2 cells following incubation for 24, 48, and 72 h. (**A**) Micrograph of TUNEL assay under bright field microscopy with darkened stains indicating DNA fragmentation within the cells. (**B**) Alteration in nuclear morphology of GNST-ITC-treated HepG2 cells evaluated using DAPI staining with arrows indicating chromatin condensation in the cell nucleus. (**C**) Fluorescence image of HepG2 cells treated with AO/PI double staining with arrows indicating viable cells (VI), chromatin condensation (CC), membrane blebbing (BL), apoptotic bodies (AB), and dead cells (DC). Results are representative of three independent experiments. Magnification ×400.

**Figure 3:**
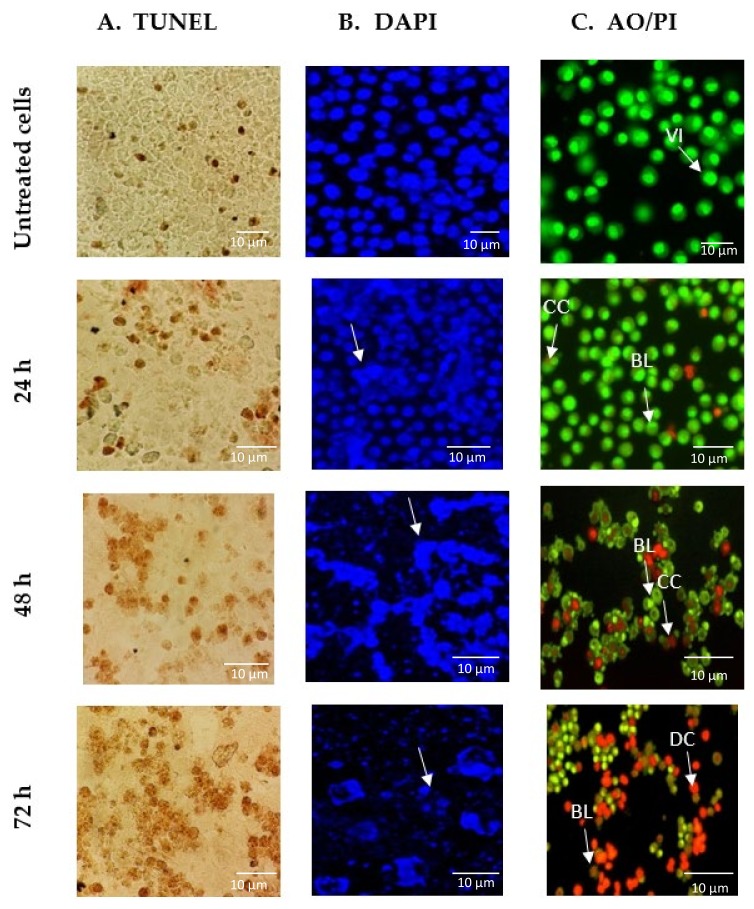
Images of GNST-ITC-induced cell death in MCF-7 cells following incubation for 24, 48, and 72 h. (**A**) Micrograph of TUNEL assay under bright field microscopy with darkened stains indicating DNA fragmentation within the cells. (**B**) Alteration in nuclear morphology of GNST-ITC-treated MCF-7 cells evaluated using DAPI staining with arrows indicating chromatin condensation in the cell nucleus. (**C**) Fluorescence image of MCF-7 cells treated with AO/PI double staining with arrows indicating viable cells (VI), chromatin condensation (CC), membrane blebbing (BL), apoptotic bodies (AB), and dead cells (DC). Results are representative of three independent experiments. Magnification ×400.

Terminal deoxynucleotidyl transferase dUTP nick end labelling (TUNEL) assay, 4′,6-diamidino-2-phenylindole (DAPI) staining, and acridine orange (AO)/ propidium iodide (PI) double staining were utilized to investigate whether GNST-ITC induced cell death due to apoptosis. A significant observation was that cells treated with GNST-ITC at IC_50_ concentration for 24, 48, and 72 h displayed clear apoptotic morphology. Using the TUNEL assay, it was observed that treatment of HepG2 and MCF-7 cells with GNST-ITC led to cell shrinkage and membrane blebbing. As shown in Figure 2A and Figure 3A, brown precipitates were evident in both cell lines. In addition, using DAPI staining to visualize changes in nucleus and formation of apoptotic bodies, which are important markers of apoptosis, it was noted that GNST-ITC induced apoptosis in both cell lines, where cells detachment, nuclear shrinkage, nuclear fragmentation, and finally formation of apoptotic bodies occurred. As shown in Figure 2B and Figure 3B, the untreated cells maintained their integrity and were colored blue. In contrast, the GNST-ITC-treated cells appeared condensed and loss of membrane integrity was apparent; these cells exhibited bright blue fluorescence, demonstrating the typical apoptotic features.

Moreover, AO/PI staining was performed to differentiate viable, early or late apoptotic, and necrotic cells under fluorescence microscopy. AO is taken up by both viable and nonviable cells emitting green fluorescence, while PI is only taken up by nonviable cells that emit red florescence. Figure 2C and Figure 3C show the intact viable cells in bright green nucleus, whereas the early apoptotic cells are indicated by a yellowish green nucleus with chromatin condensation. Orange spots in both HepG2 and MCF-7 cells show early and late apoptosis. Together, these findings demonstrate that GNST-ITC induces apoptosis in HepG2 and MCF-7 cell lines.

### 2.3. GNST-ITC-Mediated Induction of Early Apoptosis in HepG2 and MCF-7 Cell Lines

**Figure 4:**
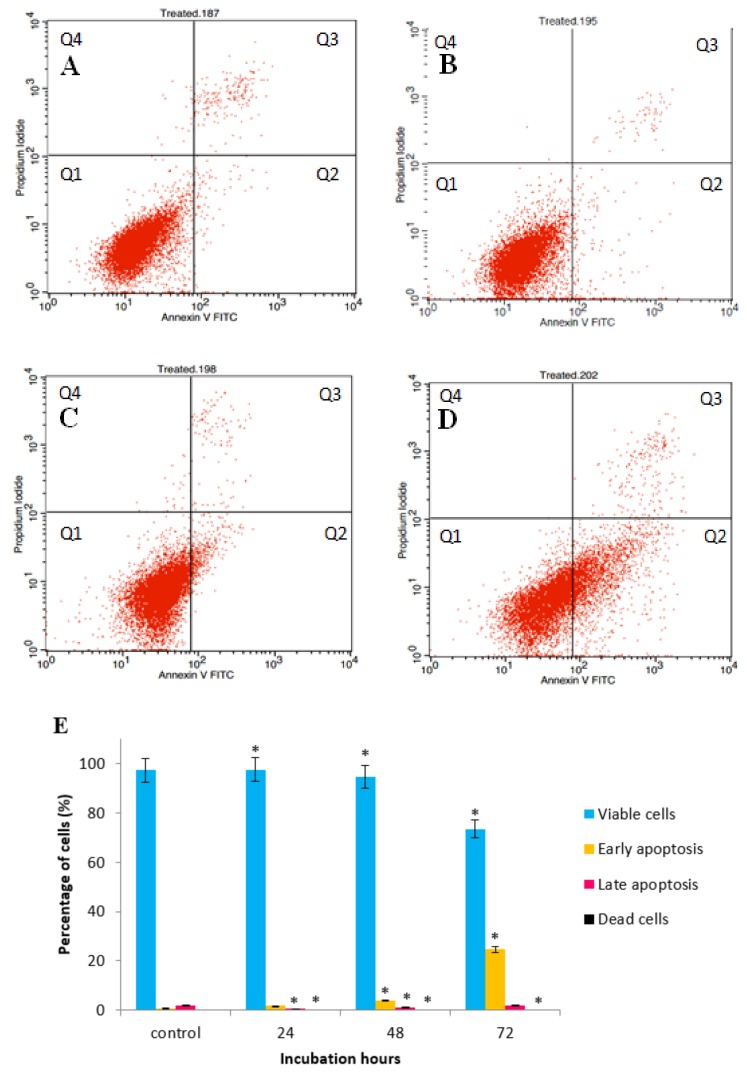
Flow cytometric analysis was performed to determine apoptotic activity in GNST-ITC-treated HepG2 cells by Annexin-V/PI double staining. HepG2 cells were treated for 24, 48, and 72 h: (**A**–**D**) control and 24 h, 48 h, and 72 h treated cells respectively. (**E**) Bar chart shows percentage of cell distribution after the treatment. Values are presented as means ± SD of triplicate experiments. Significant difference (*p* < 0.05) as compared to control is indicated by asterisk.

**Figure 5:**
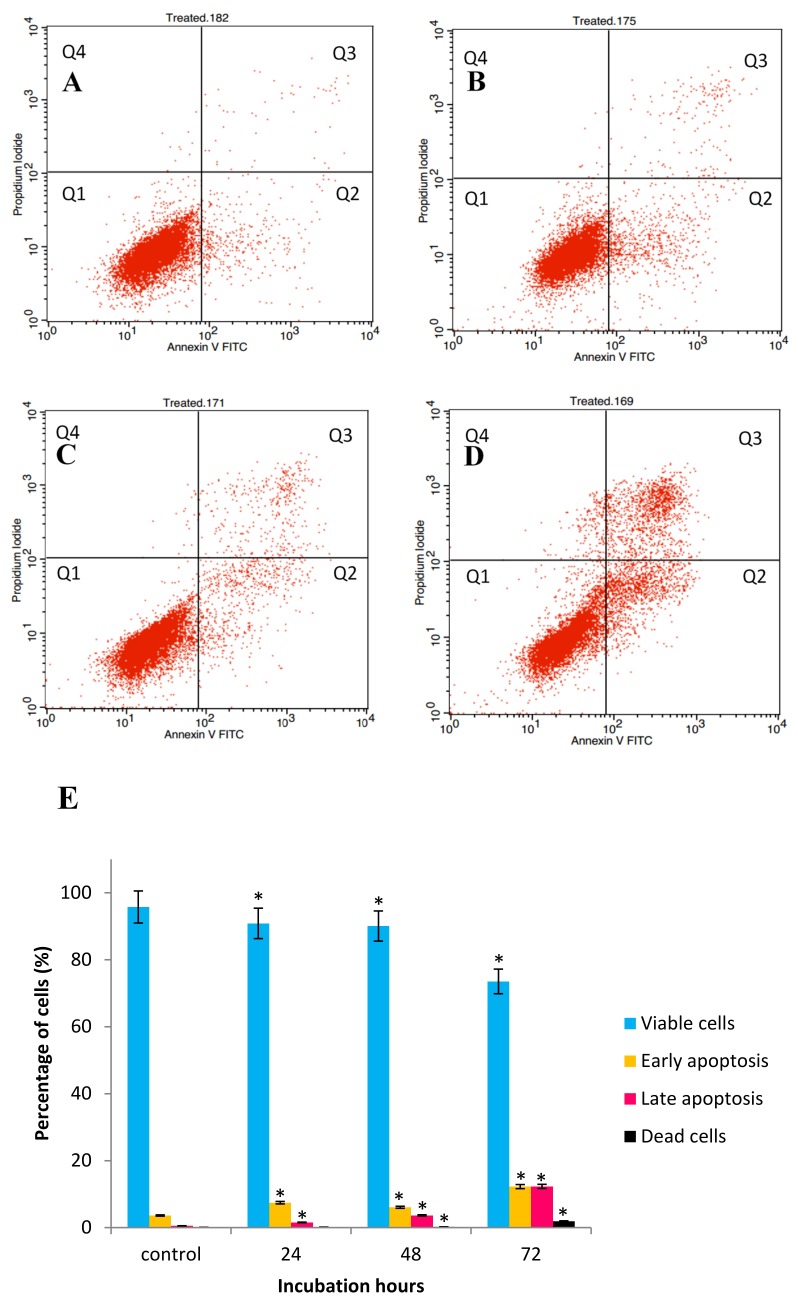
Flow cytometric analysis was performed to determine apoptotic activity in GNST-ITC-treated MCF-7 cells by Annexin-V/PI double staining. MCF-7 cells were treated for 24, 48, and 72 h: (**A**–**D**) control and 24 h, 48 h, and 72 h treated cells respectively. (**E**) Bar chart shows percentage of cells distribution after the treatment. Values are presented as means ± SD of triplicate experiments. Significant difference (*p* < 0.05) as compared to control is indicated by asterisk.

The above studies established that reduction in cell survival after exposure to GNST-ITC could be, at least partly, due to the induction of apoptosis. Externalization of phosphatidylserine is a biomarker of apoptosis that can be detected by flow-cytometry using the Annexin V-FITC apoptosis detection kit. Figure 4A–D and Figure 5A–D demonstrating the Annexin V-FITC plot analysis within four different quadrants (Q1, Q2, Q3, and Q4) represent one of triplicate experiments. There was an increase in early and late apoptosis after exposure of the cells to GNST-ITC for 24, 48, and 72 h when compared to untreated cells. The proportion of HepG2 cells that entered early apoptotic stage increased from 1.84% to 24.71% and late apoptosis from 0.66% to 1.95% as the treatment reached 72 h (Figure 4E). A similar picture emerged in MCF-7 cells (Figure 5E); at the end of 72 h, there was an increase from 7.44% to 12.25% and from 1.56% to 12.31% of early and late apoptosis, respectively.

### 2.4. GNST-ITC-Mediated Cell Cycle Arrest

**Figure 6:**
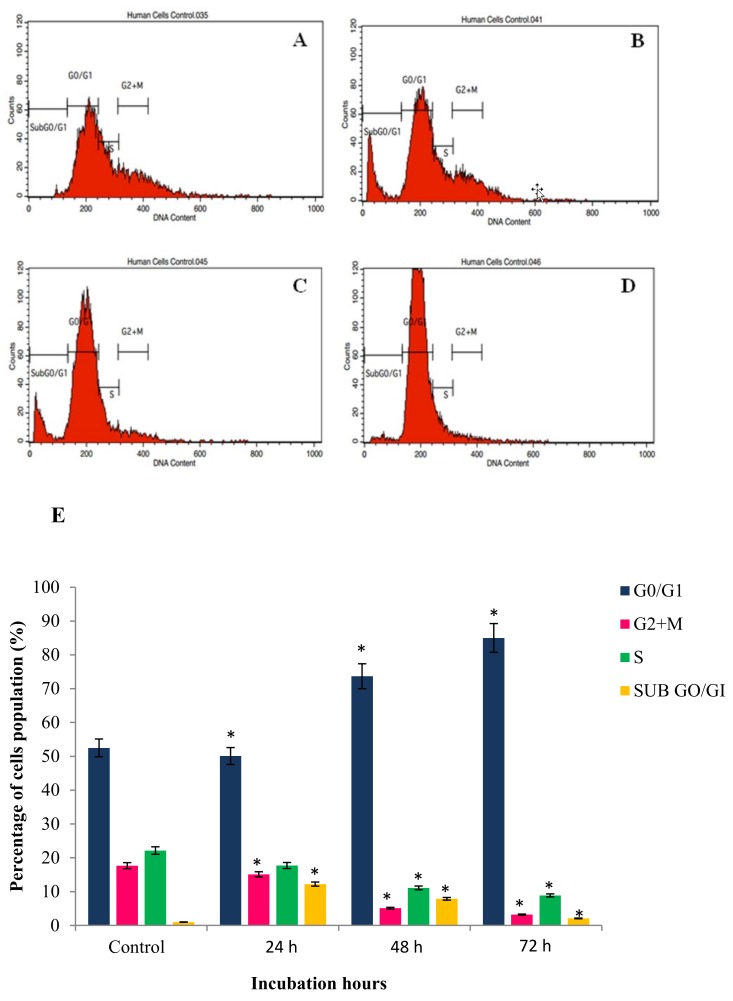
Cell cycle arrest histogram of GNST-ITC-treated HepG2 cells at 7.83 µM in a time-dependent manner by flow cytometry: (**A**–**D**) control and 24 h, 48 h, and 72 h treated cells respectively. (**E**) Bar chart shows percentage of cells distribution after the treatment. Values are presented as means ± SD of triplicate experiments. Significant difference (*p* < 0.05) as compared to control is indicated by asterisk.

**Figure 7:**
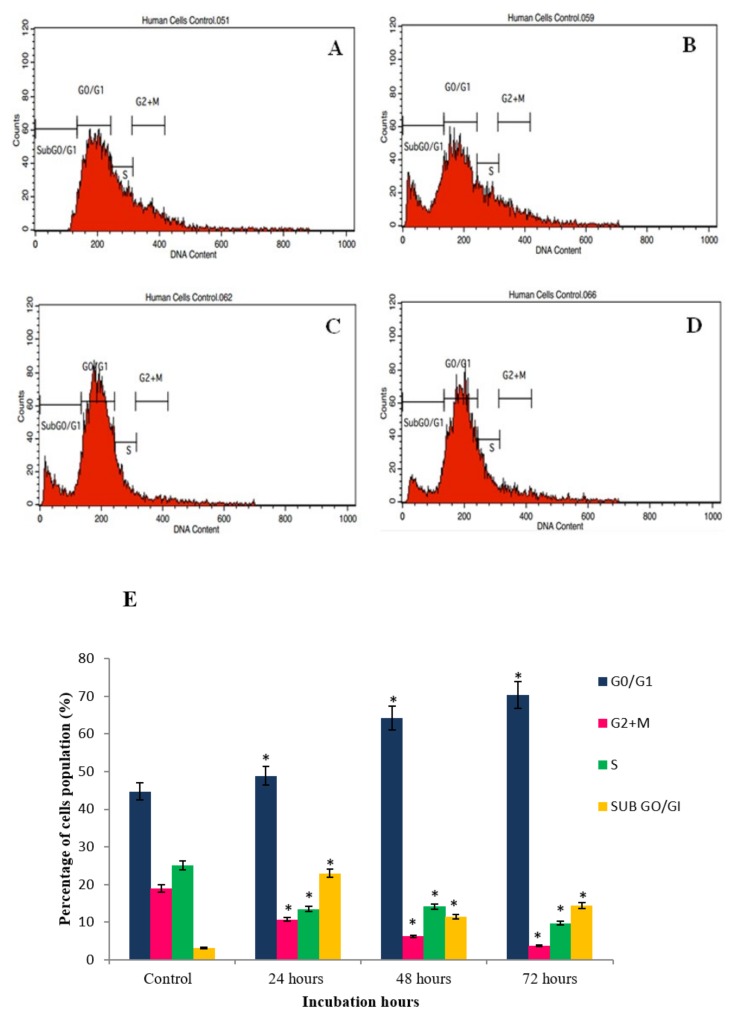
Cell cycle arrest histogram of GNST-ITC-treated MCF-7 cells at 5.02 µM in a time-dependent manner by flow cytometry: (**A**–**D**) control and 24 h, 48 h, and 72 h treated cells respectively. (**E**) Bar chart shows percentage of cells distribution after the treatment. Values are presented as means ± SD of triplicate experiments. Significant difference (*p* < 0.05) as compared to control is indicated by asterisk.

Apoptosis and cell cycle phase arrest in HepG2 and MCF-7 cancer cells were studied upon exposure to GNST-ITC at IC_50_ concentration for 24, 48, and 72 h. Flow cytometric analysis was carried out to determine cellular DNA content to establish whether growth inhibition was due to cell cycle arrest (Figure 6 and Figure 7). In HepG2 cells, treatment with GNST-ITC for 24, 48, and 72 h resulted in a time-dependent manner arrest of cell cycle in the G_2/_M phase. Similar observations were made in MCF-7 cells, where the cells were arrested in G_2/_M phase.

### 2.5. GNST-ITC-Mediated Modulation of Caspase-3/7, -8, and -9 Activities

**Figure 8:**
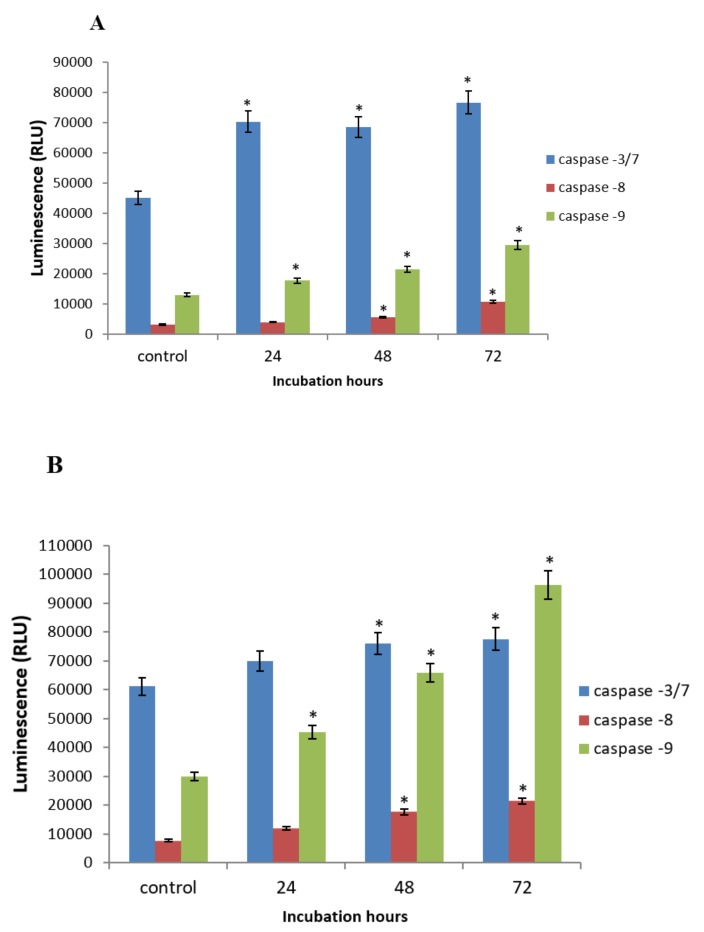
Modulation of caspase-3/7, -8, and -9 in HepG2 cells (**A**) and MCF-7 cells (**B**) treated with GNST-ITC at 7.83 μM and 5.02 μM, respectively for 24, 48, and 72 h measured using luminescence based-assay: Cells were cultured in serum free RPMI-1640 media and maintained at 37 °C and 5% CO2. Values are presented as means ± SD of triplicate experiments. Significant difference (*p* < 0.05) as compared to control is indicated by asterisk.

To evaluate the involvement of caspases in GNST-ITC-induced apoptosis, the enzymatic initiator caspases (caspase-9 and caspase-8) and effector caspase (caspase-3/7) were analyzed. Caspase-3/7 and caspase-9 activities, but not caspase-8 activity, were markedly elevated after treatment with GNST-ITC in both cell lines (Figure 8A,B).

## 3. Discussion

GNST, found abundantly in watercress, is converted into bioactive GNST-ITC and PEITC by the enzyme myrosinase upon cellular damage. PEITC has been shown to possess anticancer activity mediated by different mechanisms [10]. The apoptosis-inducing potential of GNST-ITC hydrolyzed in situ in liver and breast cancer remains to be confirmed. In the current study, GNST-ITC impaired the growth of both human hepatocellular cancer and human breast adenocarcinoma cells. The ability of GNST-ITC to inhibit the growth of these cells compares to that of tamoxifen and cisplatin, which are extensively prescribed chemotherapy agents [16]. Moreover, the study indicates that GNST-ITC-induced apoptosis involves mitochondrial dependent mechanisms.

Determination of cell viability is one critical step in assessing the cytotoxicity potential of anticancer agents. The current observation of GNST-ITC-induced cytotoxicity of HepG2 and MCF-7 is in agreement with our previous findings [17]. An IC_50_ value of 7.32 µM after exposure of MCF-7 cells to PEITC has been reported, which compares with the value (5.02 µM) obtained in the current study [18,19]. GNST-ITC was found to be a potent cytotoxic agent in HepG2 and MCF-7 cells as evidenced by a low IC_50_ value at concentration lower than 10 µM. Pharmacokinetic studies performed with sulforaphane and phenethyl isothiocyanate, in rodents, noted that such concentrations in the plasma may be achieved following exposure to doses simulating the human dietary intake [20,21].

Apoptosis or programmed cell death is a controlled cellular suicidal program to remove the damaged and dysfunctional cells while preserving the structure of surrounding tissue [22]. Apoptosis gives rise to morphological alterations during cell death such as condensation of nuclear heterochromatin and DNA fragmentation, changes in cell membrane by shrinkage, and changes in the orientation of intra cytoplasmic organelles [6]. These morphological changes serve as ideal markers for the detection of cells undergoing apoptosis and the identification of the cellular mechanisms associated to apoptosis. Based on the growth inhibition data, to further confirm the apoptotic mode of cell death, TUNEL, DAPI, and AO/PI staining were performed. GNST-ITC-treated HepG2 and MCF-7 cells displayed apoptotic morphological changes including cell shrinkage, chromatin condensation, nuclear fragmentation, and irregular shape.

Annexin V-FITC, cell cycle arrest, and caspase activities analyses were also conducted to elucidate the underpinning molecular mechanisms responsible for the observed morphological alterations in apoptotic cancer cells. Accordingly, the Annexin V-FITC assay further confirmed the observations following AO/PI staining by demonstrating in both cell lines membrane alteration via externalization of phosphatidyl serine (PS). At the early apoptotic stages, PS, which is located at the interior of lipid bilayer of the cell membrane, has high affinity towards Ca^2+^ and is translocated to the exterior of the plasma membrane; consequently, apoptotic cells can be distinguished by the presence of PS on the cell surface [23]. Therefore, cells stained with Annexin-V/FITC and PI are categorized as viable cells (lower left quadrant; Annexin^−^/PI^−^), early apoptotic cells (lower right quadrant; Annexin^+^/PI^−^), late apoptotic cell (upper right quadrant; Annexin^+^/PI^+^), and necrotic cells (upper left quadrant; Annexin^-^/PI^+^). It is thus evident that GNST-ITC induces apoptosis in both HepG2 and MCF-7 cells.

Furthermore, cell cycle progression analysis showed that GNST-ITC induced G_2_/M phase arrest in HepG2 and MCF-7 cell lines and that data from TUNEL and DAPI staining also further confirmed the DNA damage induced by GNST-ITC in apoptotic cells. The present studies established that the pattern of distribution at G_0_/G_1_ cells increased in a time-dependent manner in both cell lines. This phase arrest halts the cancer cells from entering the next phase of cell cycle, thereby inhibiting cell proliferation and proceeding towards apoptosis. It has been previously reported that PEITC induced cell cycle arrest at the G_2_/M phase in various cell lines [24,25,26], indicating the involvement of heat shock proteins (HSPs), chaperones for several client proteins involved in cell cycle control [27]. Indeed, induction of cell cycle arrest by ITCs was first reported by Hasegawa and coworkers [28], who demonstrated the accumulation of cells at G_2_/M phase after 16 h of treatment. Since then, PEITC have been found to effectively induce cell cycle arrest at different phases depending on the cell line [6].

Moreover, it was demonstrated in the present study that GNST-ITC induced in both HepG2 and MCF-7 cells involved caspase-3/7 and caspase-9 but not caspase-8. Caspase-3/7 and caspase-9 activities were increased markedly by GNST-ITC which indicates critical involvement of mitochondria in modulating pro and antiapoptotic proteins [29]. The presence of an aromatic ring on the PEITC side chain was also believed to affect intracellular reactive oxygen species (ROS) generation, leading to apoptotic and necrotic cancer cell death [30]. Lawson et al. [31] and Ahmed et al. [32] have proposed that isothiocyanate-induced inhibition of deubiquitinating enzymes (DUBs), which are linked with tumorigenesis, may also explained the potential application of isothiocyanates as a chemoprevention agent. In addition, the biocompatibility mechanism of apoptosis as well as the biomarker involved can be a useful tool for early prevention or treatment of cancer by plants containing glucosinolates [33,34].

## 4. Materials and Methods

### 4.1. Isolation and Characterisation of Gluconasturtiin (GNST)

The test compound, GNST, was purified at CREA-AA (ex CRA-CIN), Bologna, Italy and sourced through a collaborative study. The isolation and characterization of the GL were conducted according to Visentin et al. [30] and Barillari et al. [35]. In brief, 50 g of *B. verna* seeds was grounded using Ultraturrax homogeniser at medium speed for 15 min and extracted twice in boiling water (1:30 *w*/*v*) followed by centrifugation at 17,700 ×*g* for 30 min. The extract was subsequently added to 1 M Zn (OAc)_2_ in the ratio 50:1 (*v*/*v*), and the precipitated proteins were removed by centrifugation. The step was followed by loading the deproteinised extract onto a DEAE-Sephadex A-25 Pharmacia anion-exchange column (26 × 150 mm) conditioned with 25 mM acetate buffer at pH 5.6. The column was then washed with 1 L of distilled water. The elution was further carried out with 500 mL of 0.1 M K_2_SO_4_ and concentrated using a rotary evaporator to dryness at 70 °C under vacuum. Three subsequent extractions were performed with 70 mL of boiling MeOH, filtered, and concentrated to approximately 15–20% of the initial volume. The solution was warmed and gradually added drop by drop to 200 mL of pre-cooled EtOH at −20 °C until white powder was attained. Lastly, after centrifugation, the solid GNST as potassium salt was freeze-dried and sealed under vacuum. The purity of GNST was determined according to the EU official method (ISO 9167-1), based on HPLC analysis of desulfo-GLs attained through removal of the sulfate group by sulfatase-catalysed hydrolysis with sinigrin as internal standard. The analysis was conducted using a Hewlett-Packard Model 1100 HPLC system with an Inertsil ODS3 column.

### 4.2. Cell Culture

HepG2 (HB-8065, human hepatocellular carcinoma cells) and MCF-7 (HTB-22, oestrogen receptor-positive human breast adenocarcinoma cells), were obtained from American Type Culture Collection (ATCC, Manassas, VA, USA). The HepG2 and MCF-7 cells were routinely maintained and incubated at 37 °C with 5% CO2 in RPMI-1640 medium (Sigma-Aldrich, Munich, Germany), supplemented with 10% sterile-filtered fetal bovine serum (FBS) (Sigma-Aldrich, Germany) and 1% antibiotic (penicillin-streptomycin) (Sigma-Aldrich, Germany).

### 4.3. Assessment of Cytotoxicity Using the MTT Assay

Cytotoxicity test was performed by means of 3-(4,5-dimethylthiazol-2-yl)-2,5-diphenyltetrazolium bromide (MTT) assay as described by Mosmann [36]. In brief, confluent HepG2 and MCF-7 cells were plated into 96-well plates at 1 × 10^5^ cells/mL and incubated at 37 °C with 5% CO_2_ for 24 h. Cells were then treated at different concentrations of GNST (0.1–100 µM) with and without the presence of 5 µL myrosinase enzyme (Sigma Aldrich, St. Louis, MO, USA) (0.3 units/mL) as the test agent and paclitaxel and cisplatin as the positive control for 24, 48, and 72 h. At the end of the treatment, 20 µL of MTT (Sigma Aldrich, Germany) solution (5 mg/mL in PBS) was added to each well. Following incubation for approximately 4 h at 37 °C with 5% CO_2_, media containing the MTT were discarded and replaced with 100 µL of dimethyl sulfoxide (Sigma Aldrich, Germany). When the purple color developed, plates were read at 570 nm, and results were expressed as percentage of cell viability at 24, 48, and 72 h. The growth inhibition of the tested agent is expressed as the IC_50_ value.

### 4.4. Morphology Assessment of Apoptotic Cells by TUNEL Assay

DNA fragmentation of treated and untreated HepG2 and MCF-7 cells was analyzed utilizing the terminal deoxynucleotidyl transferase dUTP nick end labelling (TUNEL) assay kit (Promega, Madison, WI, USA) according to the manufacturer’s protocol. Briefly, following 24, 48, and 72 h of incubation, untreated and GNST-ITC-treated at IC_50_ concentration of HepG2 (7.83 µM) and MCF-7 (5.02 µM) cells were harvested, added onto poly-L-lysine coated slides, and fixed with 4% paraformaldehyde (Sigma-Aldrich) in PBS for 25 min at room temperature. Subsequently, the cells were permeabilized by immersing the slide in 0.2% Triton^®^ X-100 (Sigma-Aldrich) solution in PBS for 5 min. The cells were washed again in PBS twice for 5 min each; 100 μL equilibration buffer was added for 5–10 min, followed by 100 μL of terminal deoxynucleotidyl transferase (TdT) reaction mix, covered and incubated at 37 °C for 60 min. Slides were then immersed in 2× Saline Sodium Citrate (SSC) for 15 min to terminate the reactions and were washed in PBS thrice, each for 5 min, to remove unincorporated biotinylated nucleotides. Slides were subsequently immersed in 0.3% hydrogen peroxide (Sigma-Aldrich) in PBS for 3–5 min and washed again in PBS twice, each for 5 min, before adding 100 μL Streptavidin Peroxidase solution per slide and incubated for 30 min. Lastly, 100 μL of 3,3′-Diaminobenzidine (DAB) solution was added to each slide, and when the light brown background developed, the slides were examined under a light microscope (Olympus, Tokyo, Japan).

### 4.5. Morphology Assessment of Apoptotic Cells by DAPI Staining

Apoptotic cell death was also determined morphologically using 4′,6-diamidino-2-phenylindole (DAPI, Sigma-Aldrich) staining according to the method described by Ibrahim et al. [4], with slight modifications. HepG2 and MCF-7 cells were grown on sterile glass slides overnight and further incubated with GNST-ITC for 24, 48, and 72 h in a humidified atmosphere (5% CO_2_, 37 °C). Afterward, the cells were fixed with 4% paraformaldehyde and then permeabilised with Triton X-100 (0.1% in PBS). Finally, the cells were stained using DAPI in PBS (2.5 mg/mL) and viewed under fluorescence microscopy (Zeiss, Oberkochen, Germany) employing a 20× objective lens.

### 4.6. Morphology Assessment of Apoptotic Cells by AO/PI Staining

GNST-ITC-induced cell death in HepG2 and MCF-7 cells was further monitored using propidium iodide (PI) and acridine-orange (AO) double staining (Sigma-Aldrich) and examined under fluorescence microscopy according to the method of Ibrahim et al. [4] with minor modifications. Briefly, HepG2 and MCF-7 cells, plated at 1 × 10^6^ cell/mL, were treated for 24, 48, and 72 h and incubated in an atmosphere of 5% CO_2_ at 37 °C. At the end of the incubation, the cells were spun down by centrifugation at 1500 rpm for 10 min. Supernatant was discarded, and the cells were washed thrice using PBS. Fluorescent dyes containing AO (10 μg/mL) and PI (10 μg/mL) were added to the cellular pellet. The freshly stained cell suspension was dropped onto a glass slide, covered with a coverslip, and examined under fluorescence microscope within 30 min. Viable cells emit green fluorescence while maintaining their intact structure, whereas apoptotic cells exhibit a bright green nucleus showing condensation of chromatin as dense green areas. Late apoptotic cells exhibit an orange nucleus, indicating condensation of chromatin, whilst necrotic cells display an orange nucleus in the intact structure.

### 4.7. Annexin V-FITC/PI by Flow Cytometry

Quantification of apoptotic cells was carried out using the Annexin V-FITC apoptosis detection kit (Sigma-Aldrich, Israel), according to the manufacturer’s instructions. Briefly, GNST-ITC-treated cells were harvested by centrifuging at 1500 rpm for 10 min. The cell pellet was washed thrice with ice-cold PBS, resuspended with 200 µL of binding buffer (5 µL Annexin V-FITC and 195 µL binding buffer), and incubated for 15 min in the dark. Cells were once again washed and resuspended in 190 µl of binding buffer and 10 µL of PI solution and further incubated for 15 min in the dark. Stained cells were immediately analyzed through flow cytometry (Becton Dickinson, Franklin Lakes, NJ, USA).

### 4.8. Cell Cycle Analysis by Flow Cytometry

Extent of apoptosis was quantified by the reduction in DNA staining after exposure of the cells to a variety of high-affinity DNA-binding fluorochromes that intercalate into DNA. Cell cycle analysis was carried out using the CycleTEST^TM^ PLUS DNA Reagent Kit (Becton Dickinson) according to the manufacturer’s protocols. HepG2 and MCF-7 cells were seeded in 25 cm^2^ culture flasks at approximately 1 × 10^6^ cells/mL and incubated for 24 h in a humidified atmosphere of 5% CO_2_ at 37 °C. Cells were then treated with GNST-ITC for 24, 48, and 72 h; harvested; centrifuged at 1500 rpm for 10 min; and washed with PBS. Briefly, cells were resuspended in 1 mL of buffer solution, vortexed, and subsequently centrifuged at 1500 rpm for 5 min, and the supernatant was discarded. Prepared 250 μL of solution A (containing trypsin buffer) was added to the cell pellet and left to react at room temperature for 10 min. Thereafter, 200 μL of solution B (containing trypsin inhibitor and RNase buffer) was added and left again at room temperature for 10 min. Finally, 200 μL of ice-cold (2 °C to 8 °C) solution C (PI) was added to fix the cells and allowed to stand in the dark for 10 min. The percentage of cells in the G1, S, and G2 phases were analyzed by the flow cytometer (Becton Dickinson, Franklin Lakes, NJ, USA) within 30 min at room temperature.

### 4.9. Determination of Caspase-3/7, -8, and -9 Activities

Activation of caspase-3/7, -8, and -9 was assessed using a luminescence-based assay employing Caspase-Glo^TM^ 3/7, 8, and 9 assay kits (Promega Corporation, Madison, WI) according to the manufacturer’s instructions. Cells were seeded at a density of 1 × 10^6^ cells/mL in a 96-well microplate, and 50 µL of RPMI-1640 supplemented with 10% FBS was added and incubated for 24 h in a humidified atmosphere of 5% CO_2_ at 37 °C. After treatment with GNST-ITC at IC_50_ concentrations of 7.83 µM and 5.02 µM for 24, 48, and 72 h, Caspase-Glo assay reagent (100 µL) was added to each well and further incubated for 1 h at room temperature. Luminescence was measured using microplate reader (Tecan Infinite M 200 PRO, Männedorf, Switzerland).

### 4.10. Statistical Analysis

All data are expressed as the mean ± standard deviation. One-way ANOVA was used to compare the data by considering *p* < 0.05 as the level of statistical significance. All analyses were performed in triplicate (n = 3).

## 5. Conclusions

In conclusion, GNST-ITC, a hydrolysis product of GNST, exhibited potent cytotoxicity towards HepG2 and MCF-7 cells. GNST-ITC induced DNA fragmentation and nuclear condensation, which are imperative markers for apoptotic cell death. This is one of the first reports to demonstrate induction of cell cycle arrest and activation of caspase enzymes by GNST-ITC, leading to increased apoptosis. These findings serve to highlight the cancer chemopreventive potential of GNST-ITC, and further, in vivo studies using animal models of breast and liver cancer are warranted.

## Figures and Tables

**Table 1:** Cytotoxicity of gluconasturtiin-isothiocyanate (GNST-ITC) in HepG2 and MCF-7 cells.

Compound	Incubation (h)	HepG2 Cells (IC_50_)	Compound	Incubation (h)	MCF-7 Cells (IC_50_)
GNST	24	ND	GNST	24	ND
	48	ND		48	ND
	72	ND		72	ND
GNST-ITC	24	22.54 ± 0.08 µM	GNST-ITC	24	10.67 ± 0.05 µM
	48	12.14 ± 0.13 µM		48	6.19 ± 0.26 µM
	72	7.83 ± 0.34 µM		72	5.02 ± 0.08 µM
Cisplatin	24	7.78 ± 0.4 µM	Paclitaxel	24	6.92 ± 0.37 nM
	48	2.56 ± 0.44 µM		48	4.28 ± 0.77 nM
	72	1.99 ± 0.23 µM		72	3.21 ± 0.47 nM

HepG2 and MCF-7 cells were incubated with GNST (0.1–100 µM) with and without the presence of myrosinase for 24, 48, and 72 h of incubation. Cisplatin and paclitaxel served as the positive control. Results are presented as mean ± SD for triplicate determinations. ND: not detected.
